# Editorial: Corticospinal Excitability in Patients With Multiple Sclerosis

**DOI:** 10.3389/fneur.2020.635612

**Published:** 2021-01-12

**Authors:** Moussa A. Chalah, Ulrich Palm, Samar S. Ayache

**Affiliations:** ^1^EA 4391, Excitabilité Nerveuse et Thérapeutique, Université Paris-Est-Créteil, Créteil, France; ^2^Service de Physiologie - Explorations Fonctionnelles, Hôpital Henri Mondor, Assistance Publique - Hôpitaux de Paris, Créteil, France; ^3^Department of Psychiatry and Psychotherapy, Klinikum der Universität München, Munich, Germany; ^4^Medical Park Chiemseeblick, Bernau, Germany

**Keywords:** multiple sclerosis (MS), corticospinal excitability, TMS—transcranial magnetic stimulation, tDCS—transcranial direct current stimulation, non-invasive brain stimulation

A plethora of debilitating symptoms can be caused by multiple sclerosis (MS) such as sensory and motor dysfunction, cognitive impairment, mood disorder, and fatigue (1–3). Such complaints can drastically impact a patient's quality of life, are mostly assessed in a clinical manner or with the help of scales, questionnaires, or test batteries, and cannot be completely or partially relieved using pharmacotherapeutics. Moreover, their underlying mechanisms are not fully elucidated. Therefore, probing corticospinal excitability as a surrogate of the neuronal network function by using transcranial magnetic stimulation (TMS) could help in further understanding the underlying mechanisms of MS (4). TMS consists of applying a magnetic field over the scalp in a single- or double-pulse paradigm so as to obtain variables which reflect the functioning of the corticospinal system (4). These measures assess the excitability of the neuronal membrane [e.g., motor threshold (MT) and motor evoked potentials (MEP) amplitude], and the function of intracortical GABAergic and glutamatergic circuits [reflected by the intracortical inhibition (ICI) and facilitation parameters, respectively] as well as other processes [e.g., corticospinal inhibition (CSP) or interhemispheric transcallosal mechanisms; (4)]. This Research Topic focused on the exploration and modulation of corticospinal excitability in MS.

First, applying TMS could help in identifying biomarkers of the disease itself. In their perspective article, Bassi et al. reported an association between some underlying mechanisms of MS (demyelination and axonal loss) and TMS measures [e.g., low amplitudes and high latencies of MEP, high resting MT, and increased central motor conduction time (CMCT)]. In addition, an inflammation-mediated synaptopathy seems to underlie a hyperexcitability state which could appear, using TMS, as an imbalance between cortical excitatory and inhibitory processes. This could occur early in the disease process, seems to characterize relapses, and can become marked along the disease progression. Besides conventional TMS measures, new paradigms might give an additional scope on the neurophysiology of MS. For instance, by applying dual-site TMS of the ipsilateral dorsal premotor cortex (PMd) and primary motor cortex (M1), Ruiu et al. demonstrated in their original article a preserved PMd-M1 connectivity in patients with relapsing-remitting MS. Keeping this in mind, it would also be interesting to evaluate this outcome in patients with other disease phenotypes.

Second, TMS could also help in monitoring the disease evolution, especially when facing difficulties in documenting clinical progression. In their original article involving patients with progressive MS, Hardmeier et al. validated TMS-derived quantitative scores (i.e., CMCT and corticomuscular latency) that were obtained, along with clinical measures (disability and ambulation scores) over a 2-year period. Only neurophysiological measures were significantly deteriorated at 1 year, and higher effect size was obtained for neurophysiological worsening (mostly the mean corticomuscular latency) compared to clinical worsening at 2 years. These findings support the ability of neurophysiological measures to detect subtle changes before the appearance of clinically palpable progression, highlighting their potential add-on value to clinical assessment.

Third, besides the disease underlying mechanisms, MS symptoms could be explored using TMS, as reviewed by Bassi et al. For instance, anxiety was associated with interhemispheric inhibition in one study. In another study, verbal memory deficits were associated with defective short-latency afferent inhibition, a variable reflecting motor cortex cholinergic activity. In a few other works, fatigue was related to abnormal GABAergic inhibition (short-interval ICI and CSP). The latter finding was also highlighted in the review by Capone et al. who provided insight on the application of TMS, as well as other neurophysiological modalities [i.e., electroencephalography, electromyography (EMG), event related potentials, autonomic measures, and polysomnography], to explore fatigue. Capone et al. suggested the main contribution of central mechanisms to this symptom. In addition to the previous literature, Ruiu et al. described a correlation between fatigue scores and a decrease in functional connectivity during cued motor inhibition, suggesting the latter as a promising marker of MS fatigue.

Fourth, TMS measures could constitute potential outcomes for rehabilitation interventions. In the original article by Chaves et al. involving patients with progressive MS, 10 weeks of walking training resulted in significant enhancement of corticospinal excitability that was observed right after the intervention, but not 3 months later (active MT, MEP amplitude, and recruitment curve in both hemispheres; CSP in the hemisphere corresponding to the less affected hand). Some corticospinal excitability changes were significantly correlated with fatigue improvement.

Fifth, besides exploring corticospinal excitability, it is also possible to modulate it using non-invasive brain stimulation techniques such as TMS, as well as transcranial direct current stimulation (tDCS) (4, 5). Relative to TMS, tDCS is portable, easier to handle, and has a lower cost (5). As shown in the review by  Capone et al., most of the available studies employed tDCS and focused on MS fatigue. Anodal tDCS was applied over cortical areas that take part in the MS fatigue loop [i.e., left dorsolateral prefrontal cortex (DLPFC), right posterior parietal cortex, and bilateral somatosensory and/or motor cortices], and predominantly yielded significant antifatigue effects. This highlights the potential utility of this technique in managing MS fatigue. In addition to fatigue, walking and functional mobility impairments could be targeted with tDCS. This was studied by Pilloni et al. who performed a randomized, sham-controlled, proof-of-concept study in which a 20-min session of anodal M1 tDCS coupled with aerobic exercise did not result in clinical improvement (gait speed and time). This lack of effects might be attributed to the number of sessions, as the authors recently reported significant effects when performing multiple sessions (6). A third domain to target would be cognitive impairment. In their mini-review, Nasios et al. reappraised the underlying mechanisms of cognitive deficits in MS (regional tissue damage and atrophy, synaptopathy, and cognitive network dysfunction) and emphasized the need for further research to assess the effects of neuromodulation in this context. In the original article by Grigorescu et al., five consecutive daily sessions of bifrontal tDCS applied in a randomized sham-controlled manner did not ameliorate general or social cognition (i.e., attention, information processing speed, working memory, or theory of mind). Furthermore, working memory improvement (1-back test accuracy) was only observed after sham intervention, which might reflect a potential impairment of working memory following bifrontal tDCS that could be attributed to the cathode placement over the right DLPFC.

Finally, besides central non-invasive brain stimulation techniques, other approaches could be of help in harnessing neuroplasticity processes. In their mini-review, Thompson and Sinkjær presented a training method, the operant conditioning of EMG-evoked responses, as a way to enhance corticospinal excitability and consequently the motor function in MS. Via an up-conditioning training of the corticospinal system behavior, the rewarded excitability state could be learned and retained in daily life by means of iterative training. Here, one should note the relevance of assessing inflammation and cognitive status when studying the effects of this learning technique, since it relies on cognitive functions that could be halted in MS and on synaptic plasticity that could be hampered by inflammation.

Taken together, the available data suggest the promising potential of exploring and modulating corticospinal excitability for research, diagnostic, and therapeutic purposes. The current limitations arise from the low number of studies, the small sample sizes, and the interstudy variations in methods or results. There is no doubt that this field is still in its early stages of development. Therefore, future large-scale works would help in overcoming the current challenges and providing further insights on the clinical utility of this approach. A greater understanding of the neurophysiological correlates of disease characteristics and symptoms could allow for designing of patient-tailored therapies. And, combining several therapies depending on the clinical context (e.g., brain stimulation, operant conditioning of EMG-evoked responses, cognitive rehabilitation, exercise training, psychotherapies, or medications) might result in synergistic effects on the studies' outcomes.

## Author Contributions

All authors contributed to the article and approved the submitted version.

## Conflict of Interest

MC declares having received compensation from Janssen Global Services LLC. UP has a private practice with NeuroCare Group, Munich, Germany. SA declares having received travel grants or compensation from Genzyme, Biogen, Novartis, and Roche.
